# Commentary: Large-scale psychological differences within China explained by rice vs. wheat agriculture

**DOI:** 10.3389/fpsyg.2015.00950

**Published:** 2015-07-07

**Authors:** Seán G. Roberts

**Affiliations:** Language and Cognition Department, Max Planck Institute for PsycholinguisticsNijmegen, Netherlands

**Keywords:** China, rice vs. wheat, collectivism, phylogenetics, linguistics

Talhelm et al. (2014) test the hypothesis that activities which require more intensive collaboration foster more collectivist cultures. They demonstrate that a measure of collectivism correlates with the proportion of cultivated land devoted to rice paddies, which require more work to grow and maintain than other grains. The data come from individual measures of provinces in China. While the data is analyzed carefully, one aspect that is not directly controlled for is the historical relations between these provinces. Spurious correlations can occur between cultural traits that are inherited from ancestor cultures or borrowed through contact, what is commonly known as Galton's problem (Roberts and Winters, 2013). Effectively, Talhelm et al. treat the measures of each province as independent samples, while in reality both farming practices (e.g., Renfrew, 1997; Diamond and Bellwood, 2003; Lee and Hasegawa, 2011) and cultural values (e.g., Currie et al., 2010; Bulbulia et al., 2013) can be inherited or borrowed. This means that the data may be composed of non-independent points, inflating the apparent correlation between rice growing and collectivism.

The correlation between farming practices and collectivism may be robust, but this cannot be known without an empirical control for the relatedness of the samples. Talhelm et al. do discuss this problem in the supplementary materials of their paper. They acknowledge that a phylogenetic analysis could be used to control for relatedness, but that at the time of publication there were no genetic or linguistic trees of descent which are detailed enough to suit this purpose. In this commentary I would like to make two points. First, since the original publication, researchers have created new linguistic trees that can provide the needed resolution. For example, the Glottolog phylogeny (Hammarström et al., 2015) has at least three levels of classification for the relevant varieties, though this does not have branch lengths (see also “reference” trees produced in List et al., 2014). Another recently published phylogeny uses lexical data to construct a phylogenetic tree for many language varieties within China (List et al., 2014). In this commentary I use these lexical data to estimate cultural contact between different provinces, and test whether these measures explain variation in rice farming pracices. However, the second point is that Talhelm et al. focus on descent (vertical transmission), while it may be relevant to control for both descent and borrowing (horizontal transmission). In this case, all that is needed is some measure of cultural contact between groups, not necessarily a unified tree of descent. I use a second source of linguistic data to calculate simple distances between languages based directly on the lexicon. These distances reflect borrowing as well as descent.

The historical relatedness between cultures can be measured through language. Similarities and differences between languages and varieties reflect the histories of the human populations that speak them (e.g., Gray et al., 2009). Indeed, several studies have investigated the co-diffusion of language and rice growing in Austroasiatic languages (e.g., Sidwell and Blench, 2011; van Driem, 2011). For example, languages that descend from common ancestors have vocabularies with common etymologies, although the words may have changed over time to become different. Thus, languages that have close historical links tend to have more similar vocabularies and languages that have been isolated from each other for longer tend to have more dissimilar vocabularies. This measure may be a proxy for the transmission of cultural practices or knowledge.

Similarly, speakers borrow words from other languages and varieties meaning that varieties that are geographically close tend to be more similar. This may also be a proxy for the adoption of other cultural traits, such as farming practices (or, indeed, collectivism, though Minkov, 2012 argues that certain cultural values cannot be borrowed directly).

This data could be used in two ways. First, the linguistic similarities between cultures could be used as a statistical control for non-independence in the synchronic data. The relationship between rice growing and collectivism would be robust if it persists when removing the variance explained by shared history. Secondly, one could use the linguistic data to look at diachronic changes. It is possible to reconstruct the historical contingencies between cultural groups using language data and phylogenetic techniques (e.g., Dunn et al., 2011). This involves reconstructing past changes as cultural groups come into contact and divide. Support for Talhelm's theory would come from demonstrating that when the prevalence of rice growing increased, so did the estimated collectivism measure. The estimated historical relations could be integrated with what is known about the actual historical spread of farming practices (e.g., Kovach et al., 2007; Fuller, 2011; Dodson et al., 2013).

This kind of quantitative study is beyond the scope of this commentary, but it is at least possible to test whether rice-growing exhibits historical contingencies. I use a comparison of the vocabularies of the languages spoken in each province as a proxy for historical relations. A list of vocabulary and cognate coding for basic concepts for (Han) varieties within China were taken from List et al. (2014) (the cognate coding was derived automatically, see List et al. for details). The average distance between varieties was computed as the number of shared cognates (words which are related by descent) between the two varieties (calculation done using *LingPy*, List and Moran, 2013). Varieties were associated with the provinces in which their data was collected. The distance between two provinces was calculated as the average of the distance between each pair of varieties within each province. In this way, a matrix of distances was produced which represented how dissimilar the vocabularies of each pair of provinces was.

A second distance matrix was produced which represented the difference in the proportion of rice growing between each pair of provinces (taken from Talhelm et al.). Complete data was available for 40 language varieties from 18 provinces. The rice and linguistic distances were compared using a Mantel test (which compares the correlation between two matrices with the distribution of the correlation when one of the matrices is permuted). The difference in rice growing was significantly correlated with the linguistic distance measure (*r* = 0.27, *p* = 0.02, 10,000 permutations). That is, provinces which are more similar in the proportion of rice growing are also more similar in their vocabularies.

The same test was done using linguistic data from the ASJP database (Wichmann et al., 2013). Languages with origins within China were identified from the Ethnologue catalog (Lewis et al., 2015) along with the provinces where each was mainly spoken (these include languages associated with many ethnic groups, including Han). The distances between vocabularies were computed as the normalized edit distances (LDND) used in Wichmann et al. (2011). Given two words from different varieties for the same concept, the edit distance is the number of changes it takes to convert one word to the other, normalized by maximum number of possible edits. Similar words have a lower distance. The distance between varieties was taken as the average distance between each word in the vocabulary. The LDND measure further normalizes the distance between varieties by eliminating meaning-specific variation (see Wichmann et al.). Distances between provinces were calculated as above. Data were available for 139 languages from 18 provinces. Again, the differences in proportion of rice growing correlated significantly with the difference in vocabulary (*r* = 0.38, *p* = 0.0002, 10,000 permutations).

A tree of historical relations can be estimated from the historical distances using hierarchical clustering. Figure 1 shows this tree (from the first dataset) projected onto a map of China. The “rice–wheat” border from Talhelm et al. is highlighted. This separates the high rice production areas in the south from low rice production areas in the north. It's clear that the historical relations align with this border. Indeed, the root of the tree splits provinces in the north from those in the south and sub-branches of the tree spread east-to-west.

**Figure 1:**
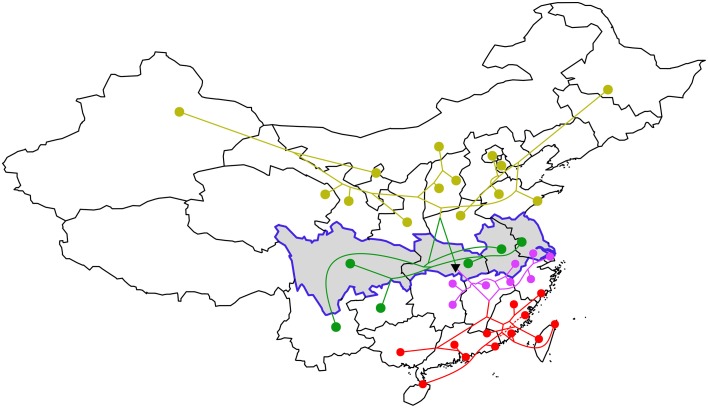
**A tree of historical relationships between language varieties constructed from hierarchical clustering of similarities in vocabulary**. Provinces on the “rice–wheat” border are highlighted in gray (provinces south of the border produce more rice). The black triangle indicates the root of the tree. The top four branches of the tree are colored differently (varieties within the same branch are more closely related than varieties in different branches). Branch lengths are not meaningful.

These results suggest that the prevalence of rice growing is related to cultural contact. This means that a more careful consideration of historical relationships between provinces is warranted before the link between rice production and collectivism can be confirmed. One part of Talhelm et al.'s study which may be more robust to the findings presented here are the results at the county-level for neighboring provinces. This analysis uses more fine-grained groups than the varieties used in this paper. However, cultural contact can also be assessed at the level of accent and dialect (e.g., Spruit et al., 2009). Further surveys similar to Talhelm et al.'s may consider eliciting linguistic data from participants, as well as psychological or sociological data, with the aim of using them as controls for relatedness.

In general, researchers should be wary of correlations that do not control for shared cultural history. On a more positive note, as an increasing amount of data becomes available, cultural and linguistic data can be used together with other sources to investigate human history.

## Conflict of interest statement

The author declares that the research was conducted in the absence of any commercial or financial relationships that could be construed as a potential conflict of interest.
